# Cancer targeting by TCR gene-engineered T cells directed against Kita-Kyushu Lung Cancer Antigen-1

**DOI:** 10.1186/s40425-019-0678-x

**Published:** 2019-08-28

**Authors:** Bridget Marcinkowski, Sanja Stevanović, Sarah R. Helman, Scott M. Norberg, Carylinda Serna, Benjamin Jin, Nikolaos Gkitsas, Tejas Kadakia, Andrew Warner, Jeremy L. Davis, Lisa Rooper, Christian S. Hinrichs

**Affiliations:** 1 Experimental Transplantation and Immunology Branch, National Cancer Institute, 10 Center Drive, Room 4B04, Bethesda, MD 20892 USA; 2 Pathology and Histology Laboratory, Frederick National Laboratory for Cancer Research, Frederick, MD 21702 USA; 3 Surgical Oncology Program, National Cancer Institute, Bethesda, MD 20892 USA; 4 Johns Hopkins School of Medicine, Baltimore, MD 21205 USA

**Keywords:** Gastric cancer, Breast cancer, KK-LC-1, Cell therapy, Gene therapy, T cell receptor, CAR-T, Gene engineering, T cell, Immunotherapy

## Abstract

T cell receptor (TCR) gene-engineered T cells have shown promise in the treatment of melanoma and synovial cell sarcoma, but their application to epithelial cancers has been limited. The identification of novel therapeutic TCRs for the targeting of these tumors is important for the development of new treatments. Here, we describe the preclinical characterization of a TCR directed against Kita-Kyushu Lung Cancer Antigen-1 (KK-LC-1, encoded by *CT83*), a cancer germline antigen with frequent expression in human epithelial malignancies including gastric cancer, breast cancer, and lung cancer. Gene-engineered T cells expressing the KK-LC-1 TCR (KK-LC-1 TCR-Ts) demonstrated recognition of *CT83*+ tumor lines in vitro and mediated regression of established *CT83+* xenograft tumors in immunodeficient mouse models. Cross-reactivity studies based on experimental determination of the recognition motifs for the target epitope did not demonstrate cross-reactivity against other human proteins. *CT83* gene expression studies in 51 non-neural tissues and 24 neural tissues showed expression restricted exclusively to germ cells. *CT83* was however expressed by a range of epithelial cancers, with the highest expression noted in gastric cancer. Collectively, these findings support the further investigation and clinical testing of KK-LC-1 TCR-Ts for gastric cancer and possibly other malignancies.

**Electronic supplementary material:**

The online version of this article (10.1186/s40425-019-0678-x) contains supplementary material, which is available to authorized users.

## Introduction

Cellular therapy with antigen receptor gene-engineered T cells that express chimeric antigen receptors (CARs) or T cell receptors (TCRs) is a promising approach to cancer treatment. T cells that express CARs (CAR-Ts) have demonstrated efficacy in the treatment of leukemia and lymphoma [1, 2]. T cells that express TCRs (TCR-Ts) have shown clinical activity in melanoma and synovial cell sarcoma [3]. However, success with this approach in epithelial cancers has been limited [4].

One constraint has been the identification of tumor-restricted antigens and of receptors that target these antigens [5]. Kita-Kyushu Lung Cancer Antigen-1 (KK-LC-1, encoded by *CT83*) is a cancer germline (CG) antigen that is reported to have restricted expression in healthy tissues and frequent expression in certain epithelial cancers including lung cancer, gastric cancer, and breast cancer [6–8]. Furthermore, it is the only member of its family, and therefore might be targeted without risk of intra-family cross-reactivity. Hence, KK-LC-1 appears to be an attractive target for antigen receptor gene therapy [4].

Most CG antigen genes map to chromosome X, and expression is regulated by epigenetic mechanisms that often result in coordinate gene expression. *CT83* is located at Xq23, distinct from other CG antigens including *MAGE* gene family members and *CTAG1A* (also known as *NY-ESO-1*) [9]. KK-LC-1 was identified as a potential immunotherapy antigen by characterization of the target of a lung adenocarcinoma-reactive T cell clone [6].

We identified a KK-LC-1-reactive T cell receptor (KK-LC-1 TCR) from the tumor-infiltrating lymphocytes (TILs) of a patient with cervical cancer who had a complete tumor response to TIL therapy [10]. Here we report the preclinical evaluation of the receptor, including targeting of tumor cells in vitro, regression of xenograft tumors in vivo, cross-reactivity studies, and assessment of antigen expression by healthy tissues and tumors. These findings form the basis for a clinical trial for patients with wide-ranging metastatic epithelial cancers.

## Results

**Fig. 1:**
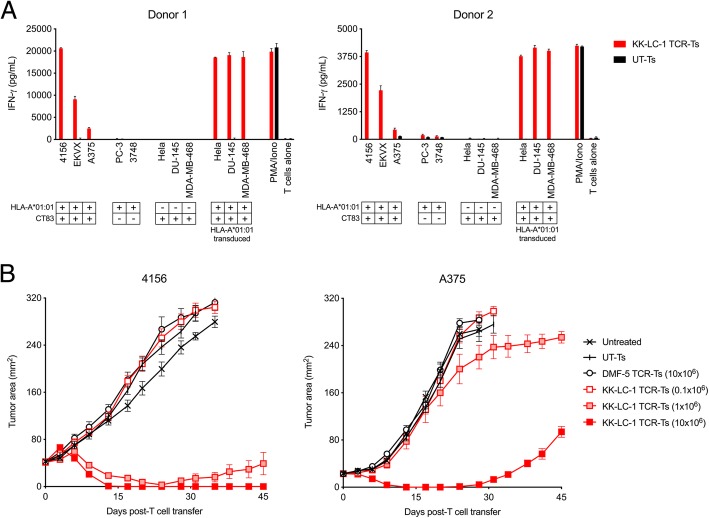
KK-LC-1 TCR-Ts display tumor recognition in vitro and mediate tumor regression in vivo. **a** Human CD8+ T cells from each of 2 donors were transduced to express the KK-LC-1 TCR (KK-LC-1 TCR-Ts) or were not transduced (UT-Ts). Tumor recognition was tested in an overnight coculture assay with the target cell line indicated on the x-axis. The quantity of IFN-γ in the culture supernatants was determined by ELISA. Expression of *CT83* and HLA-A*01:01 by each target cell line is indicated in the key below the x-axis. HLA-A*01:01 transduced cell lines were *CT83+* and transduced with a γ-retrovirus to express HLA-A*01:01. “PMA/Iono” indicates T cells that were stimulated with PMA and ionomycin. “T cells alone” indicates T cells that were cultured without target cells or stimulation. **b** KK-LC-1 TCR-Ts or control T cells indicated in the figure legend were administered intravenously to NSG mice bearing established 4156 or A375 subcutaneous tumors (as indicated above each graph). Serial tumor measurements were plotted at the timepoints indicated on the x-axis. Untreated mice did not receive any therapy. UT-Ts were not transduced. DMF-5 TCR-Ts target an irrelevant antigen (melanoma associated antigen-1) [12]. *N* = 10 mice per group. Error bars indicate the standard error of the mean. This experiment was performed twice with similar results

The KK-LC-1 TCR targets KK-LC-1_52-60_ presented by the HLA-A*01:01 molecule [10]. Predicted binding of KK-LC-1_52-60_ to other HLA molecules was weaker (Additional file 1: Table S1) [11]. We tested if third-party human T cells that were transduced to express the KK-LC-1 TCR (KK-LC-1 TCR-Ts) recognized tumor cell lines that express *CT83* and HLA-A*01:01 in vitro. In overnight coculture assays, KK-LC-1 TCR-Ts from 2 donors displayed interferon (IFN)-γ release in response to cell lines that expressed the target antigen and the HLA restriction element, which indicated recognition of these lines (Fig. 1a, Additional file 1: Figure S1). These included the unmanipulated cell lines 4156 (cervical cancer), EKVX (lung cancer), and A375 (melanoma). All tested cell lines that expressed both the target antigen and the restriction element were recognized; conversely, all cell lines that did not express both the target antigen and the restriction element were not recognized.

To assess if systemically administered KK-LC-1 TCR-Ts could mediate tumor responses in vivo, we employed a murine xenograft model for the treatment of subcutaneous, established 4156 or A375 tumors. A single intravenous injection of KK-LC-1 TCR-Ts induced regression of 4156 tumors (Fig. 1b). At the highest dose (10 × 10^6^ cells) all mice demonstrated complete tumor regression. A375 tumors, which display heterogenous CT83 expression (Additional file 1: Figure S2a and b), eventually recurred, and recurrent tumors showed low CT83 expression (Additional file 1: Figure S2c), which may have contributed to their late relapse. Nonetheless, all mice with either 4156 or A375 tumors treated with at least 1x10^6 KK-LC-1 TCR-Ts displayed tumor regression. These data indicate that KK-LC-1 TCR-Ts can target tumor cells in vitro and can mediate the regression of tumors in vivo.

**Fig. 2:**
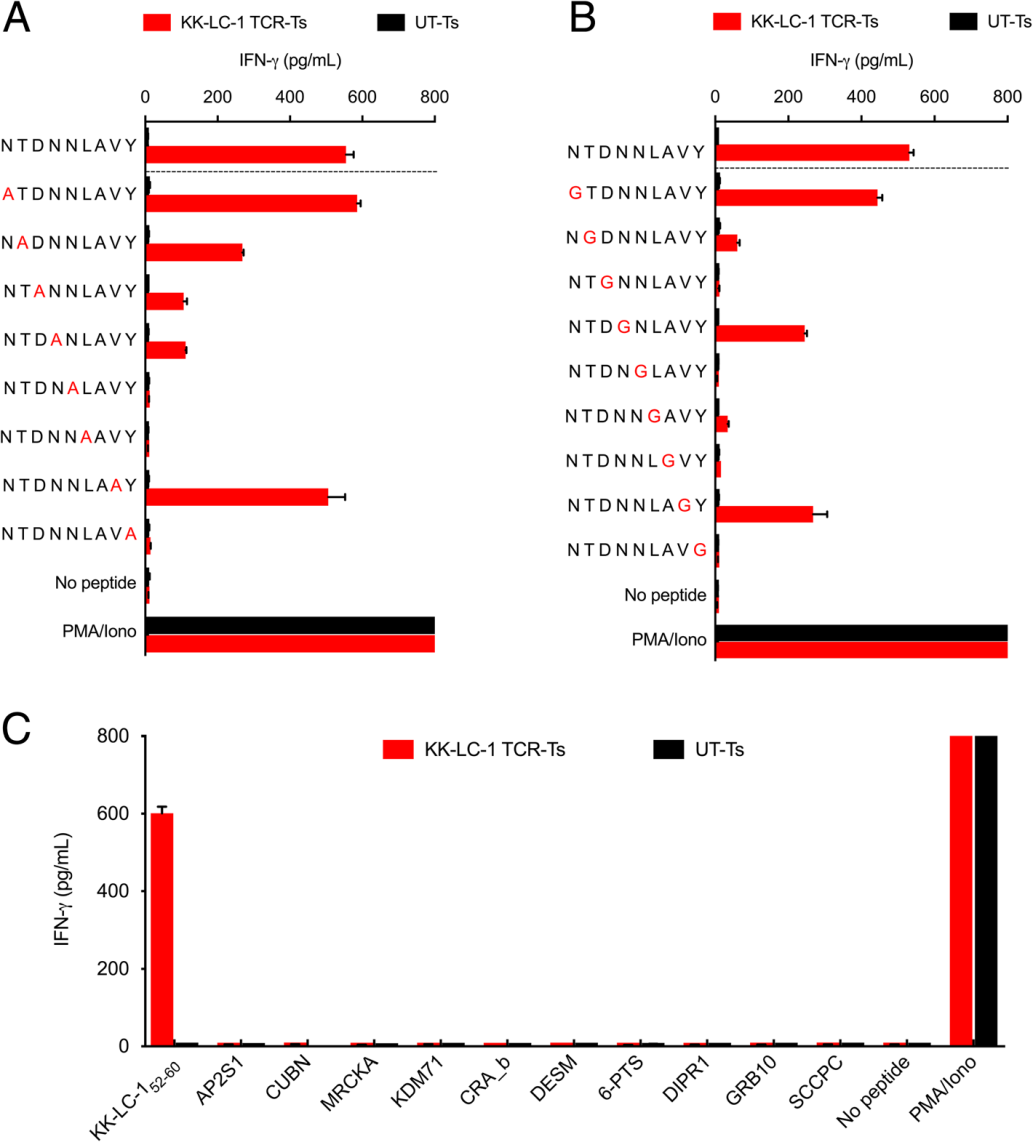
KK-LC-1 TCR-Ts did not demonstrate cross-reactivity with peptides derived from other human proteins. The IFN-γ production assays shown were performed by coculture of KK-LC-1 TCR-Ts with autologous EBV-LCLs loaded with 1 μg/mL of the peptide indicated. Coculture supernatants were harvested after overnight coincubation. IFN-γ concentration was determined by ELISA. Error bars represent the SD of 2 technical replicates. The “no peptide” conditions had target cells without peptide. “PMA/Iono” indicates T cells that were stimulated with PMA and ionomycin. “UT-Ts” were untransduced control T cells from the same donor as the KK-LC-1 TCR-Ts. **a** To guide cross-reactivity testing, alanine scanning of KK-LC-1_52-60_ was performed. An alanine residue was substituted for the native residue at each position of KK-LC-1_52-60_. **b** To compliment alanine substitution and assess the influence of position 7 on target recognition, glycine scanning also was performed. **c** Peptides derived from human proteins that demonstrated identity at the contact residues inferred by the experiments in (**a**) and (**b**) or by a BLAST search for candidate peptides that shared at least 5/9 residues (55% identity) were tested for KK-LC-1 TCR-T recognition

We next evaluated KK-LC-1 TCR-Ts for cross-reactivity against potential epitopes of other human proteins. To determine which residues in the KK-LC-1_52-60_ epitope were critical for recognition by the KK-LC-1 TCR, we performed alanine and glycine scanning of the KK-LC-1_52-60_ peptide. Alanine substitutions at positions 3, 4, 5, 6, and 9 and glycine substitutions at positions 2, 3, 5, 6, 7, and 9 caused a greater than 75% decrease in IFN-γ release as compared to the wild type peptide. Based on these data, the residues at positions 3, 5, 6, and 7 were inferred to be the most essential non-anchor residues for TCR recognition (Fig. 2a and b). The ScanProsite online tool was used to search for human proteins that shared these positions (Additional file 1: Table S2) [13]. In addition, a Basic Local Alignment Search Tool (BLAST) search identified 6 more human peptides with high levels of sequence identity to KK-LC-1_52-60_ (Additional file 1: Table S2). KK-LC-1 TCR-Ts were tested for recognition of the 10 candidate peptides in a coculture assay; recognition was not detected (Fig. 2c). Thus, the KK-LC-1 TCR did not demonstrate detectable cross-reactivity against human peptides in vitro*.*

**Fig. 3:**
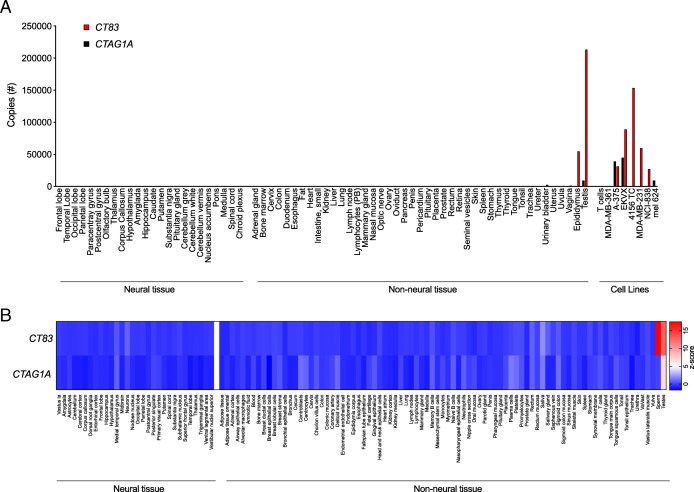
*CT83* expression is limited in healthy tissue to immune-privileged or non-vital sites. **a** cDNA from a custom-made panel of 51 non-neural tissues and 24 neural tissues was assessed for *CT83* and *CTAG1A* expression by qRT-PCR. cDNA from cancer cell lines and human T cells were included as controls. The y-axis displays total copy number. This experiment was performed twice with similar results. **b** Healthy tissue expression of *CT83* and *CTAG1A* is graphed as a heatmap based on data collected from the U133plus2 Affymetrix microarray available through BioGPS. The values shown are z-scores produced by the barcode function of the R package “frma.” z-scores greater than 5 are suggestive of gene expression in the tissue

Targeting of an antigen that is expressed by healthy tissues with TCR-T therapy can result in severe autoimmune toxicity [5]. To determine if *CT83* is expressed by healthy tissues, we performed quantitative reverse transcription polymerase chain reaction (qRT-PCR) on a custom array of cDNA from healthy tissues. Because other members of the CG antigen family have been found to be expressed at low levels in the brain we included a range of neural tissues in the screening panel [14]. *CT83* expression was detected in positive control samples of epididymis and testis, which lack HLA expression and thus, cannot be targeted by T cells. *CT83* was not detected in other tissues except at a very low level (< 2500 copies) in urinary bladder (Fig. 3a). To further interrogate healthy tissues for expression of *CT83*, we queried the BioGPS database (Barcode on normal tissues dataset) (Fig. 3b) [15]. Expression of *CT83* did not exceed a z-score of 5, the value that suggests expression in a given tissue, except in sperm and testis. *CTAG1A*, the gene that encodes Cancer/testis antigen 1, an antigen that has been targeted with TCR-Ts without reactivity against healthy tissues, displayed a similar pattern of expression. Taken together, these data suggest that *CT83* expression by healthy tissues is restricted to germ cells.

**Fig. 4:**
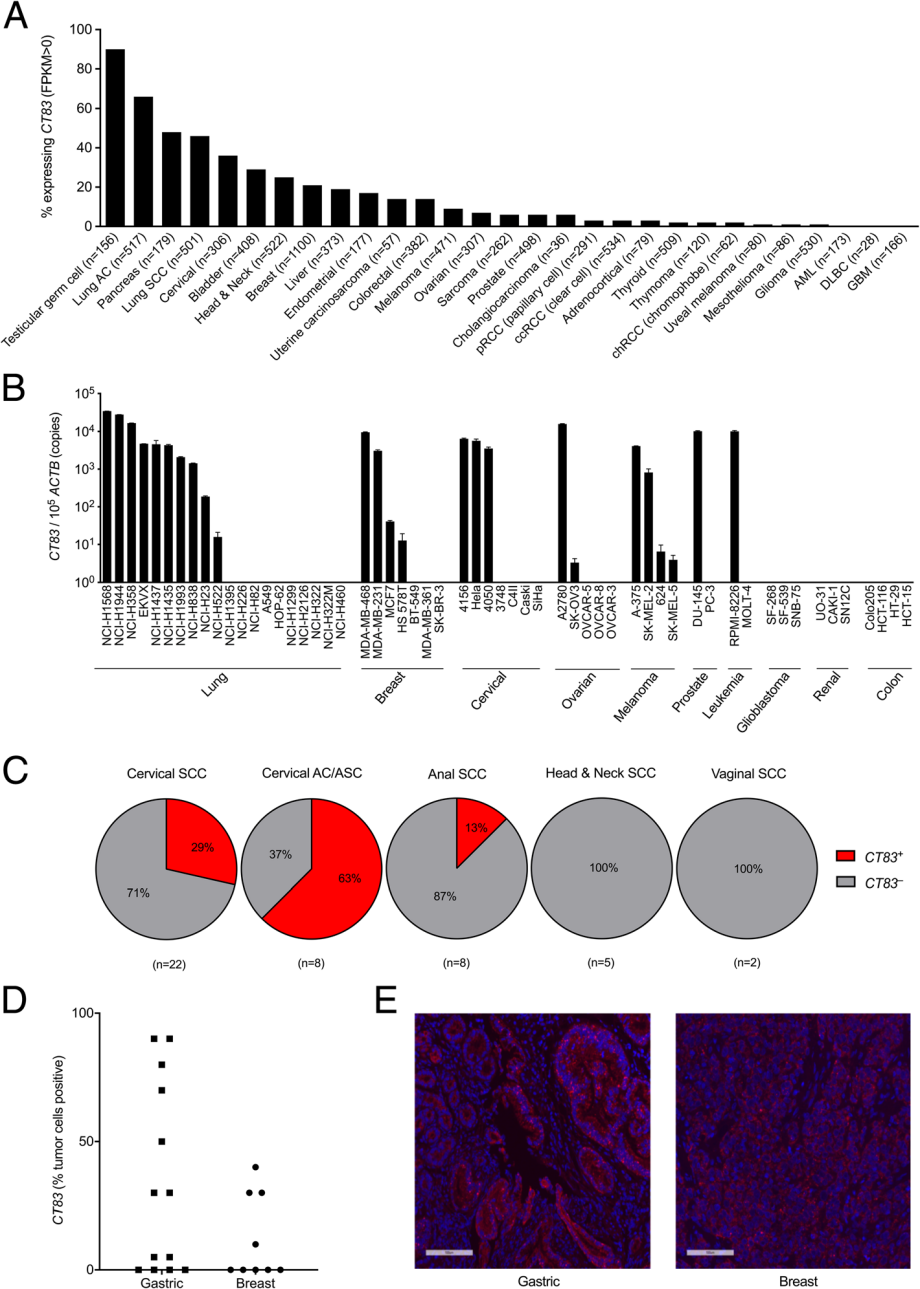
*CT83* expression by cancer cell lines and tumors. **a** The frequency (% of tumors that express the antigen) of *CT83* expression by different cancer types was assessed. Expression data are derived from TCGA Provisional dataset accessed through cBioportal. Fragments Per Kilobase of transcript per Million mapped reads (FPKM) values > 0 were considered positive. The number of samples per cancer type is indicated in parentheses. **b** A panel of cancer cell lines was assessed for *CT83* expression by qRT-PCR. The y-axis displays *CT83* copies per 10^5^ copies of *ACTB*. This experiment was performed twice with similar results. **c** The frequency of HPV+ metastatic cancers that express *CT83* was assessed by qRT-PCR. The number of samples per cancer type is indicated in parentheses. Experiments were performed twice. **d** Intratumoral heterogeneity of *CT83* expression was assessed by RNA ISH using RNAScope. The tumor type is indicated on the x-axis. The frequency of *CT83*+ tumor cells was scored by an independent, blinded pathologist. **e** Sample images of *CT83*+ tumors by RNAScope are shown. Magnification is 20X. Nuclei were counterstained with DAPI (blue)

KK-LC-1 expression has been reported in gastric cancer, triple negative breast cancer, and lung adenocarcinoma [6–8]. To investigate whether KK-LC-1 is expressed in other cancer types, we tested 57 cell lines from 10 different types of cancer for CT83 expression by qRT-PCR. Lung, breast, cervical, ovarian, melanoma, prostate, and leukemia cancer cell lines were found to express *CT83*, albeit with varying levels and frequencies of expression (Fig. 4a). Bioinformatic analysis of The Cancer Genome Atlas (TCGA) Provisional data set accessed on the cBioPortal Cancer Genomics public database also indicated *CT83* expression in a wide range of cancers, with more frequent expression (> 20% of tumors) in testicular cancer, lung adenocarcinoma, pancreatic cancer, lung squamous cell carcinoma, cervical cancer, bladder cancer, head and neck cancer, and breast cancer (Fig. 4b). We previously observed *CT83* expression in a human papillomavirus (HPV) + metastatic cervical cancer. Examination of a bank of metastatic cervical cancer specimens revealed expression in 6/21 (29%) of cervical squamous cell carcinomas and 5/8 (63%) of cervical adenocarcinomas (Fig. 4c). In other HPV+ cancers, expression was detected in 1/8 anal cancers, 0/5 head and neck cancers, and 0/2 vaginal cancers (Fig. 4c). To assess the frequency of cells within a tumor that express *CT83*, we performed RNA in situ hybridization with RNAScope on gastric cancers, breast cancers, and lung cancers. The highest frequency of positive cells occurred in gastric cancers, of the 13 samples tested, 9 were positive for *CT83* expression (median: 50%, range: 5 to 90%). Triple negative breast cancer also had varying frequencies of expression, with 4/9 samples positive for *CT83* (Fig. 4d and e). Non-small cell lung cancer and pancreatic cancer were also assessed but expressed the antigen less frequently and demonstrated a lower fraction of positive cells (range: 0 to 5%). These data suggest that gastric cancer may be a favorable disease in which to target KK-LC-1 and that other cancers may be appropriate but in fewer patients.

## Discussion

Here, we describe the characterization of a TCR for the targeting of cancers that express the cancer germline antigen KK-LC-1. T cells engineered to express this TCR displayed specific recognition of KK-LC-1+ tumor lines in vitro and mediated regression of KK-LC-1+ tumors in vivo. KK-LC-1 TCR-Ts did not demonstrate cross-reactivity against human proteins that share contact residue motifs with the intended target. The gene encoding KK-LC-1, *CT83*, was not expressed by healthy human tissues other than germ cells. It was expressed, however, by diverse types of epithelial cancers at variable frequencies and with heterogenous intratumoral expression levels. Expression was highest in gastric cancer, where by RNA in situ hybridization (ISH) 9/13 samples were positive, and 5 displayed expression in at least 50% of tumor cells.

Autoimmune toxicity from unintended cross-reactivity of TCR-Ts against healthy tissues has prevented the development of otherwise promising TCR-T therapies [16–18]. KK-LC-1 TCR-Ts did not display cross-reactivity against human protein epitopes that shared recognition motifs or substantial sequence identity. The cross-reactivity testing based on alanine and glycine scanning to identify TCR contact residues may not identify all potential cross-reactive peptides; a full scan of all amino acid substitutions at each position may be more sensitive [19]. Despite this, the likelihood of KK-LC-1 TCR cross-reactivity against human proteins is relatively low as it was subjected to human thymic selection, and the complementarity-determining regions were not altered. Severe autoimmune TCR-T-mediated toxicity has also resulted from the targeting of antigens that are expressed by healthy tissues [12, 20]. *CT83* does not appear to be expressed by vital human tissues, as it was not detected in a panel of 51 non-neural (except germ cells) and 24 neural tissues by qRT-PCR. It is important to note that due to differences in mouse and human major histocompatibility complex molecules, safety cannot be assessed by the animal models in this study. In addition, data that xenograft models, such as those employed in this work, can predict treatment efficacy in humans is lacking. Thus, a phase I clinical trial with careful dose escalation will be required.

KK-LC-1 appears to be an attractive target antigen for TCR-T therapy as it is frequently expressed by a range of epithelial cancers, and it is not expressed by vital healthy tissues. ISH revealed varying intratumoral heterogeneity of expression, which has been observed with other CG antigen targets and may be an important consideration in the selection of types of cancer and specific patients to treat with this approach. Gastric cancers commonly demonstrated *CT83* expression, and a high fraction of cell expressed the antigen in some tumors (5/9 tumors examined showed at least 50% positivity). Taken together, these data support the continued study of KK-LC-1 TCR-Ts for the treatment of gastric cancer and possibly other epithelial malignancies.

## Materials and methods

### Animal care and in vivo experiments

Animal research protocols were approved by the NIH Animal Use and Care Committee. NSG mice (The Jackson Laboratory) were housed in NIH facilities. Tumors were established by subcutaneous injection of 1 × 10^7^ 4156 cells or 4 × 10^6^ A375 cells. Seven days after tumor cell injection, mice were treated with a single dose of cells administered by tail vein injection. Tumor size was measured with calipers and is reported as tumor area (mm^2^).

### Cell lines

Tumor cell lines were obtained from ATCC and the NCI’s Division of Cancer Treatment and Diagnosis Tumor Repository, except 4156, 4050, and 3748 which were generated in our laboratory. Tumor cell lines were grown in culture media based on RPMI 1640, IMDM, or DMEM (Thermo Fisher Scientific) with 10% fetal bovine serum (HyClone). Cell line identity was confirmed by morphology, HPV E6 and E7 expression, and *CT83* expression. HLA class I typing was determined by the NIH Clinical Center HLA Laboratory or by review of publicly available records. All cell lines were checked regularly for mycoplasma. 293-A*01:01 cell lines were generated by transduction of 293 cells with a bicistronic retrovirus encoding HLA-A*01:01 and truncated CD34. Transduced cells were selected by cell separation based on CD34 (Miltenyi Biotec).

### Quantitative reverse transcription polymerase chain reaction

To assess expression of *CT83*, RNA was extracted from the cancer cell lines and HPV+ metastatic cancers using RNeasy Plus Micro Kit (Qiagen). RNA concentration and purity was assessed by NanoDrop spectrophotometer (Thermo Fisher Scientific). 1 μg of RNA was then used to generate cDNA using qScript cDNA Supermix (Quanta Bio). Expression of the genes of interest was determined by qRT-PCR with Taqman primer/probe sets (Thermo Fisher Scientific) specific for the *CT83* gene (Hs02386421_g1,), *CTAG1A/B gene* (Hs00265824_m1)*,* and the housekeeping *ACTB* gene (Hs99999903_m1) using the Quantstudio 3 RT-PCR system (Applied Biosystems) according to manufacturer’s standard instructions. Serially diluted DNA plasmids of CT83 and ACTB were used to generate standard curves for copy number quantification using standard procedures. The thermal cycling conditions used were as follows: 95 °C 7 min; 95 °C 15 s, 60 °C 30 s × 40 cycles; 4 °C. A detailed protocol for qRT-PCR can be found in the Additional file 1.

### Retroviral transduction of T cells

Peripheral blood mononuclear cells (PBMCs) were isolated from healthy human volunteers and transduced with a retroviral vector encoding the KK-LC-1 TCR as previously described [10]. Briefly, the 293GP packaging cell line was transfected with the plasmid of interest (pMSGV1-TCR) and the pRD114 envelope plasmid using Lipofectamine 2000 (Life Technologies). Retroviral supernatant was harvested 48 h later and used to transduce PBMCs that had been stimulated with soluble 50 ng/mL anti-CD3 (OKT3, Miltenyi Biotec) and 300 IU/mL rhIL-2 (Prometheus) for 2 days prior to retroviral transduction. Transduction efficiency was determined by flow cytometric analysis using the anti-mouse TCRβ-chain antibody. Detailed protocols for retroviral supernatant production and for retroviral transduction of T cells can be found in the Additional file 1.

### Flow cytometry

Fluorescently-conjugated antibodies were purchased from BD Biosciences (anti-human CD4-FITC, clone SK3; anti-human CD8-PE-Cy7, clone SK1), Biolegend (anti-human CD3-BV421, clone SK7), and eBioscience (anti-human CD34-APC, clone 4H11; anti-mouse TCRβ-chain-PE, clone H57–597). Flow cytometry was conducted with a Novocyte (Acea Biosciences) and analyzed using FlowJo software (TreeStar Inc). In all analyses, doublets and dead cells were gated out using propidium iodide (Sigma Aldrich) and forward and side scatter. CD3+ cells were gated on before examining the population of interest. This gating strategy is depicted in Additional file 1: Figure S3.

### Immunological assays

Antigen recognition assays were performed by overnight coincubation of effector cells with target cells. Readout for these co-cultures was the production of IFN-γ as determined by enzyme-linked immunosorbent assay (ELISA) (R&D Systems). For tumor recognition testing, 6 × 10^4^ KK-LC-1 TCR-Ts or an equal number of control cells were cocultured with 1 × 10^5^ tumor cells. For cross-reactivity testing, 8 × 10^4^ KK-LC-1 TCR-Ts or an equal number of control cells were cocultured with 8 × 10^4^ Epstein Barr Virus-transformed lymphoblastoid cell lines (EBV-LCLs) pulsed with 1 μg of peptide. Peptides were synthesized by GenScript. As a positive control, T cells were stimulated with 50 ng/mL phorbol 12-myristate 13-acetate (PMA; Sigma) and 500 ng/mL ionomycin (Sigma).

### In silico search

The ScanProsite tool was used to perform searches for human peptides that contain the potential KK-LC-1_52-60_ TCR recognition motifs identified by alanine and glycine scanning. Searches were performed with motifs that included matches at positions 3, 5, 6, and 7 (X-X-D-X-N-L-A-X-X).

NCI protein BLAST was used to identify additional non-KK-LC-1 peptides within the human genome with a high level of sequence identity to KK-LC-1_52-60_. Peptides greater than 9 residues or less than 8 residues were excluded. All candidate peptides that shared at least 5/9 residues (55% identity) were tested for recognition in vitro. The BLAST and ScanProsite search parameters were adjusted as previously described [16].

### Chromogenic in situ hybridization (CISH)

*CT83* detection by CISH was performed with the 2.5 LS Reagent Kit - Red (RNAscope) using the Bond RX System (Leica Biosystems) to hybridize *CT83*-specific probes (RNAscope 2.5 LS Probe- Hs-CT83-O1) (ACD) to the target mRNA. Homo sapiens peptidylprolyl isomerase B (cyclophilin B) (*PPIB*) was used as a positive control, and a bacterial gene (dihydrodipicolinate reductase (*dapB*)) was used as negative control. Human non-small cell lung cancer (including adenocarcinoma, squamous cell carcinoma, and large cell), and triple negative breast cancer samples provided by the Cooperative Human Tissue Network which is funded by the National Cancer Institute (NCI). Other investigators may have received specimens from the same subjects. Human gastric adenocarcinoma samples were obtained from the Surgical Oncology Program of the NCI. ISH staining and imaging were performed by the Molecular Pathology Lab of the Frederick National Laboratory for Cancer Research. Slides were digitized using Aperio ScanScope FL Scanner (Leica Biosystems). *CT83* expression was manually quantified by a anatomic pathologist (LMR) based on the presence of punctate nuclear and cytoplasmic signals within tumor cells.

### Analysis of predicting binding KK-LC-1_52-60_ to MHC-I molecules

The MHCI binding predictions were made using the IEDB analysis resource Consensus tool [11], which combines predictions from ANN aka NetMHC (4.0) [21–23], SMM [24] and Comblib [25]. The following parameters were used: Prediction Method- IEDB recommended 2.19; MHC sources species- human; HLA Class I allele reference set [26].

### Analysis of gene expression data from bioinformatic repositories

The public database BioGPS was used to analyze antigen expression in normal tissue. The Barcode on Normal Tissues dataset (U133plus2 Affymetrix microarray) was selected and *CT83* (probeset: 1559258_a_at) and *CTAG1A* (probeset: 211674_x_at) expression data were extracted. For *CTAG1A*, multiple probesets were available and one was selected based on the lowest levels of background. The database cBioportal was accessed to analyze *CT83* expression in cancer. All expression data were derived from TCGA Provisional dataset.

### Statistical analysis

Statistical tests were performed using GraphPad Prism 7 Software.

## Additional file

Additional file 1: Supplemental Methods. **Table S1.** Predicted binding of KK-LC-152-60 to MHC-I molecules. **Table S2.** Peptides identified by an in silico search and tested for cross-reactivity. **Figure S1.** Determination of HLA-A*01:01 expression in transduced cell lines by flow cytometry. **Figure S2.** CT83 expression levels differed in the cell lines used for in vivo experiments. **Figure S3.** Sample gating strategy for flow cytometry. (DOCX 15400 kb)

## Data Availability

The datasets used and/or analyzed during the current study are included in this published study or are available from the corresponding author on reasonable request.

## Abbreviations

BLAST: Basic Local Alignment Search Tool
CARs: Chimeric antigen receptors
CG: cancer germline
EBV-LCLs: Epstein Barr Virus-transformed lymphoblastoid cell lines
ELISA: enzyme-linked immunosorbent assay
IFN: interferon
ISH: in situ hybridization
KK-LC-1: Kita-Kyushu Lung Cancer Antigen-1
PBMC: peripheral blood mononuclear cells
PMA: phorbol 12-myristate 13-acetate
qRT-PCR: quantitative reverse transcription polymerase chain reaction
TCGA: The Cancer Genome Atlas
TCR: T cell receptor
TILs: tumor-infiltrating lymphocytes

## References

[1] Brudno JN, Kochenderfer JN (2018). Chimeric antigen receptor T-cell therapies for lymphoma. Nat Rev Clin Oncol.

[2] Salter AI, Pont MJ, Riddell SR (2018). Chimeric antigen receptor-modified T cells: CD19 and the road beyond. Blood..

[3] Robbins PF, Kassim SH, Tran TLN, Crystal JS, Morgan RA, Feldman SA (2015). A pilot trial using lymphocytes genetically engineered with an NY-ESO-1-reactive. Clin Cancer Res Off J Am Assoc Cancer Res..

[4] Hinrichs CS (2016). Molecular pathways: breaking the epithelial Cancer barrier for chimeric antigen receptor and T-cell receptor gene therapy. Clin Cancer Res Off J Am Assoc Cancer Res.

[5] Hinrichs CS, Restifo NP (2013). Reassessing target antigens for adoptive T-cell therapy. Nat Biotechnol.

[6] Fukuyama T, Hanagiri T, Takenoyama M, Ichiki Y, Mizukami M, So T (2006). Identification of a new cancer/germline gene, KK-LC-1, encoding an antigen recognized by autologous CTL induced on human lung adenocarcinoma. Cancer Res.

[7] Shida A, Futawatari N, Fukuyama T, Ichiki Y, Takahashi Y, Nishi Y (2015). Frequent high expression of Kita-Kyushu lung Cancer Antigen-1 (KK-LC-1) in gastric Cancer. Anticancer Res.

[8] Paret C, Simon P, Vormbrock K, Bender C, Kolsch A, Breitkreuz A (2015). CXorf61 is a target for T cell based immunotherapy of triple-negative breast cancer. Oncotarget..

[9] O’Leary NA, Wright MW, Brister JR, Ciufo S, Haddad D, McVeigh R (2016). Reference sequence (RefSeq) database at NCBI: current status, taxonomic expansion, and functional annotation. Nucleic Acids Res.

[10] Stevanovic S, Pasetto A, Helman SR, Gartner JJ, Prickett TD, Howie B (2017). Landscape of immunogenic tumor antigens in successful immunotherapy of virally induced epithelial cancer. Science..

[11] Kim Y, Ponomarenko J, Zhu Z, Tamang D, Wang P, Greenbaum J (2012). Immune epitope database analysis resource. Nucleic Acids Res.

[12] Johnson LA, Morgan RA, Dudley ME, Cassard L, Yang JC, Hughes MS (2009). Gene therapy with human and mouse T-cell receptors mediates cancer regression and targets normal tissues expressing cognate antigen. Blood..

[13] de Castro E, Sigrist CJA, Gattiker A, Bulliard V, Langendijk-Genevaux PS, Gasteiger E (2006). ScanProsite: detection of PROSITE signature matches and ProRule-associated functional and structural residues in proteins. Nucleic Acids Res.

[14] Chen Y-T, Scanlan MJ, Venditti CA, Chua R, Theiler G, Stevenson BJ (2005). Identification of cancer/testis-antigen genes by massively parallel signature sequencing. Proc Natl Acad Sci U S A.

[15] McCall MN, Uppal K, Jaffee HA, Zilliox MJ, Irizarry RA (2011). The gene expression barcode: leveraging public data repositories to begin cataloging the human and murine transcriptomes. Nucleic Acids Res.

[16] Cameron BJ, Gerry AB, Dukes J, Harper JV, Kannan V, Bianchi FC (2013). Identification of a titin-derived HLA-A1-presented peptide as a cross-reactive target for engineered MAGE A3-directed T cells. Sci Transl Med.

[17] Linette GP, Stadtmauer EA, Maus MV, Rapoport AP, Levine BL, Emery L (2013). Cardiovascular toxicity and titin cross-reactivity of affinity-enhanced T cells in myeloma and melanoma. Blood..

[18] Morgan Richard A., Chinnasamy Nachimuthu, Abate-Daga Daniel, Gros Alena, Robbins Paul F., Zheng Zhili, Dudley Mark E., Feldman Steven A., Yang James C., Sherry Richard M., Phan Giao Q., Hughes Marybeth S., Kammula Udai S., Miller Akemi D., Hessman Crystal J., Stewart Ashley A., Restifo Nicholas P., Quezado Martha M., Alimchandani Meghna, Rosenberg Avi Z., Nath Avindra, Wang Tongguang, Bielekova Bibiana, Wuest Simone C., Akula Nirmala, McMahon Francis J., Wilde Susanne, Mosetter Barbara, Schendel Dolores J., Laurencot Carolyn M., Rosenberg Steven A. (2013). Cancer Regression and Neurological Toxicity Following Anti-MAGE-A3 TCR Gene Therapy. Journal of Immunotherapy.

[19] Border EC, Sanderson JP, Weissensteiner T, Gerry AB, Pumphrey NJ (2019). Affinity-enhanced T-cell receptors for adoptive T-cell therapy targeting. Oncoimmunology..

[20] Parkhurst MR, Yang JC, Langan RC, Dudley ME, Nathan D-AN, Feldman SA (2011). T cells targeting carcinoembryonic antigen can mediate regression of metastatic colorectal cancer but induce severe transient colitis. Mol Ther J Am Soc Gene Ther.

[21] Nielsen M, Lundegaard C, Worning P, Lauemoller SL, Lamberth K, Buus S (2003). Reliable prediction of T-cell epitopes using neural networks with novel sequence representations. Protein Sci Publ Protein Soc.

[22] Lundegaard C, Lamberth K, Harndahl M, Buus S, Lund O, Nielsen M (2008). NetMHC-3.0: accurate web accessible predictions of human, mouse and monkey MHC class I affinities for peptides of length 8-11. Nucleic Acids Res.

[23] Andreatta M, Nielsen M (2016). Gapped sequence alignment using artificial neural networks: application to the MHC class I system. Bioinforma Oxf Engl.

[24] Peters B, Sette A (2005). Generating quantitative models describing the sequence specificity of biological processes with the stabilized matrix method. BMC Bioinformatics.

[25] Sidney J, Assarsson E, Moore C, Ngo S, Pinilla C, Sette A (2008). Quantitative peptide binding motifs for 19 human and mouse MHC class I molecules derived using positional scanning combinatorial peptide libraries. Immunome Res.

[26] Weiskopf D, Angelo MA, de Azeredo EL, Sidney J, Greenbaum JA, Fernando AN (2013). Comprehensive analysis of dengue virus-specific responses supports an HLA-linked protective role for CD8+ T cells. Proc Natl Acad Sci U S A.

