# Supplementary material for: Cancer targeting by TCR gene-engineered T cells directed against Kita-Kyushu Lung Cancer Antigen-1
Source: J Immunother Cancer. 2019 Aug 28;7:229. doi: 10.1186/s40425-019-0678-x (PMC6712783; doi:10.1186/s40425-019-0678-x)
Supplement: Supplementary file 1 — Supplemental Methods. Table S1. Predicted binding of KK-LC-152-60 to MHC-I molecules. Table S2. Peptides identified by an in silico search and tested for cross-reactivity. Figure S1. Determination of HLA-A*01:01 expression in transduced cell lines by flow cytometry. Figure S2. CT83 expression levels differed in the cell lines used for in vivo experiments. Figure S3. Sample gating strategy for flow cytometry. (DOCX 15400 kb) [file 40425_2019_678_MOESM1_ESM.docx]

**Supplementary Materials for**

Cancer Targeting by TCR Gene Engineered T Cells Directed against Kita-Kyushu Lung Cancer Antigen-1

Bridget Marcinkowski^1^, Sanja Stevanović^1^, Sarah Helman^1^, Scott M. Norberg^1^, Carylinda Serna^1^, Benjamin Jin^1^, Nikolaos Gkitsas^1^, Tejas Kadakia^1^, Andrew Warner^2^, Jeremy L. Davis^3^, Lisa Rooper^4^, Christian S. Hinrichs^1*^

^*^Corresponding author: Christian S. Hinrichs, M.D. (hinrichs@mail.nih.gov)

**This supplementary file includes:**

Protocol for qRT-PCR

Protocol for retroviral supernatant production

Protocol for retroviral transduction of T cells

Table S1 and S2

Figure S1, S2, and S3

**Expanded Methods**

**Protocol for qRT-PCR**

To assess expression of *CT83*, RNA was extracted from the cancer cell lines and HPV+ metastatic cancers using RNeasy Plus Micro Kit (Qiagen). RNA concentration and purity were assessed by NanoDrop spectrophotometer (Thermo Fisher Scientific). 1 ug of RNA was then used to generate cDNA using qScript cDNA Supermix (Quanta Bio). Expression of the genes of interest was determined by qRT-PCR with Taqman primer/probe sets (Thermo Fisher Scientific) specific for the *CT83* gene (Hs02386421_g1), *CTAG1A/B gene* (Hs00265824_m1)*,* and the housekeeping *ACTB* gene (Hs99999903_m1) using the Quantstudio 3 RT-PCR system (Applied Biosystems) according to manufacturer’s standard instructions. Serially diluted DNA plasmids of *CT83* and *ACTB* were used to generate standard curves for copy number quantification using standard procedures. The required volumes for the reaction were calculated using the following table.

|  | **Final Concentration** | **20 μl rxn** |
| --- | --- | --- |
| PCR grade water up to 20 μl |  | As required |
| qPCR Master Mix (2X) | 1X | 10 μl |
| Forward Primer (10 μM) | 100 - 400 nM | Variable |
| Reverse Primer (10 μM) | 100 - 400 nM | Variable |
| Probe | 100 - 500 nM | Variable |
| Template DNA or cDNA | <250 ng | Variable |

The mix was pipetted into wells and the plate was sealed, briefly spun, and run using the following thermal cycling conditions:

1. Denaturation: 95°C 7 min

2. Denaturation: 95°C 15

3. Annealing and Elongation: 60°C 30 s

4. Repeat steps 2 and 3 for 40 cycles

5. Hold at 4°C.

**Protocol for retroviral supernatant production**

Day 1:

293GP cells were counted and plated in DMEM, 10% FCS media with standard additives.

| **Plate** | **# cells/well (*10^6^ )** | **media /well (mL)** |
| --- | --- | --- |
| 6-well plate | 0.8 | 2 |
| 100 mm plates | 4 | 10 |
| 150 mm plates | 10 | 22.5 |

Day 2:

The media was replaced with DMEM, 10% FCS without antibiotics 30 minutes prior to transfection.

| **Plate** | **media per well (mL)** |
| --- | --- |
| 6-well plate | 1.5 |
| 100 mm plates | 10 |
| 150 mm plates | 22.5 |

Mix A and Mix B were prepared in non-polystyrene tubes and incubated separately for 5 minutes at room temperature.

| **MIX A** | **6-well plate** | **10 cm^2^ plates** | **15 cm^2^ plates** | **Plates (#) _____** |
| --- | --- | --- | --- | --- |
| Retroviral Vector plasmid (µg)  Conc: __________________ | 1.5 | 9 | 20.25 |  |
|  | ____  µL | ____    µL | ____    µL |  |
| RD114 (envelope plasmid) (µg)  Conc: __________________ | 0.75 | 4 | 10.13 |  |
|  | 0.4 µL | 2.1 µL | 5.4 µL |  |
| Opti-MEM (mL) (Life Technologies) | 0.25 | 1.5 | 3.4 |  |

| **MIX B** | **6-well plate** | **10 cm^2^ plates** | **15 cm^2^ plates** | **Plates (#) _____** |
| --- | --- | --- | --- | --- |
| Lipofectamine (2000) (µL)  (Life Technologies) | 10 | 60 | 135 |  |
| Opti-MEM (mL) (Life Technologies) | 0.25 | 1.5 | 3.4 |  |

Mix A and Mix B were mixed together gently and incubated for 20 minutes at room temperature. The mix was added dropwise to the plates containing the 293GP cells and gently swirled.

| **Plate** | **Add per well (mL)** | **Total volume (mL)** |
| --- | --- | --- |
| 6-well plate | **0.5** | 2 |
| 100 mm plates | **3** | 13 |
| 150 mm plates | **6.75** | 29.25 |

The cells were incubate at 37^0^C for 6-8 hrs. Then the media was changed to fresh DMEM, 10% FCS no antibiotics.

| **Plate** | **Change media per well (mL)** |
| --- | --- |
| 6-well plate | 2 |
| 100 mm plates | 10 |
| 150 mm plates | 22.5 |

The cells were then incubated at 37^0^C for 48 hrs (from time of transfection).

Day 4:

The retroviral supernatant was harvested (48 hr harvest). Media was added again as above for an additional 24 hrs. The cells were centrifuged and the supernatant was filtered through a 0.22µm filter, aliquoted, and stored at 80^o^C.

Day 5:

The 72 hr retroviral supernatant was harvested and collected again as described above.

**Protocol for retroviral transduction of T cells**

Day 1:

Donor PBMCs were thawed and plated at a concentration of 0.5*10^6^ cells per 24-wells well in 2 mL of T cell media media containing 300 IU/mL rhIL-2 (Chiron) + 50 ng/mL anti-CD3 (OKT3, Miltenyi Biotec).

Day 2:

Non-tissue culture 24-well plates were coated with Retronectin (Takara) at final concentration of 20 µg/ml in PBS. 500 µL was added per well. The plates were wrapped and incubated overnight at 4 ^o^ C.

Day 3:

The Retronectin solution was aspirated and the plates were blocked with 2 mL of 2% BSA in      PBS for 30 minutes at room temperature. The plates were washed 2x with 1 mL/well of PBS. The PBS was aspirated at 0.5 mL/well  of retroviral supernatant was added. The plates were spun at 2000xg for 2 hrs at 32 ^o^ C. After spinning, the supernatant was aspirated and 0.125-0.25x10^6^T cells/well in 1 ml of T cell media containing 300 IU/mL IL-2 were added. The plates were spun at 1500 RPM for 10 minutes. The plates were incubated at 32 ^o^ C overnight.

Day 4:

The transduced cells were transferred to a tissue-culture 24-well plate.

Day 6:

Transduction efficiency was assessed by flow cytometry using an anti-mouse TCRβ-chain-PE, clone H57-597 antibody (eBioscience).

**Supplemental Tables:**

**Table S1: Predicted binding of KK-LC-1_52-60_ to MHC-I molecules.**

| Allele | Percentile Rank | ann_ic50 (nM) |
| --- | --- | --- |
| HLA-A*01:01 | 0.11 | 3.77 |
| HLA-A*30:02 | 0.9 | 130.5 |
| HLA-A*26:01 | 1.03 | 2531.54 |
| HLA-B*35:01 | 7.3 | 1005.41 |
| HLA-A*68:01 | 8.6 | 2473.86 |
| HLA-A*11:01 | 10.85 | 6179.86 |
| HLA-B*57:01 | 13.5 | 11722.02 |
| HLA-B*15:01 | 15 | 7719.34 |
| HLA-A*03:01 | 18.5 | 12776.34 |
| HLA-B*58:01 | 21 | 9250.01 |
| HLA-A*32:01 | 24 | 20079.5 |
| HLA-B*44:02 | 29.5 | 29104.2 |
| HLA-B*53:01 | 36 | 25228.8 |
| HLA-A*33:01 | 39 | 33385.11 |
| HLA-A*02:06 | 39.5 | 15892.39 |
| HLA-B*44:03 | 39.5 | 39480.02 |
| HLA-A*31:01 | 44 | 22453.01 |
| HLA-A*23:01 | 45 | 36066.05 |
| HLA-B*40:01 | 45.5 | 24474.35 |
| HLA-A*68:02 | 48 | 27960.58 |
| HLA-B*07:02 | 50 | 25955.33 |
| HLA-A*02:01 | 52 | 28549.36 |
| HLA-A*30:01 | 60 | 23931.77 |
| HLA-A*02:03 | 62 | 29495.09 |
| HLA-B*08:01 | 62 | 23753 |
| HLA-A*24:02 | 68.5 | 42919.38 |
| HLA-B*51:01 | 76 | 29458.4 |

*The IEDB Analysis Resource was used to bioinformatically predict the binding of KK-LC-1_52-60_ to frequently occurring MHC-I alleles. The alleles are listed according to percentile rank and artificial neural network IC_50_ (ann_ic50). Generally, peptides with IC_50_ values <50 nM are considered high affinity, <500 nM intermediate affinity, and <5000 nM low affinity [11].*

**Table S2: Peptides identified by an in silico search and tested for cross-reactivity.**

| Human protein | Peptide |
| --- | --- |
| KK-LC-1_52-60_ | N-T-D-N-N-L-A-V-Y |
| Adaptor protein-2 complex subunit sigma (AP2S1) | V-N-D-N-N-L-A-Y-l |
| Cubilin (CUBN) | D-G-D-N-N-L-A-Q-Q |
| Serine/threonine-protein kinase MRCK alpha (MRCKA) | T-L-D-N-N-L-A-T-E |
| Lysine-specific demethylase 7A (KDM71) | P-T-D-E-N-L-A-R-Y |
| Pregnancy-induced growth inhibitor (CRA_b) | V-T-D-N-H-L-T-V-Y |
| Desmin (DESM) | Y-T-D-N-W-L-A-V-Y |
| 6-pyruvoyl tetrahydrobiopterin synthase (6-PTS) | E-T-D-N-N-I-V-V-Y |
| Dihydropyrimidinase-related protein 1 (DIPR1) | N-T-E-D-D-L-P-V-Y |
| Growth factor receptor-bound protein (GRB10) | N-T-A-N-N-L-D-V-P |
| Small-conductance Ca^2+^-activated potassium channel (SCCPC) | N-N-S-N-N-L-A-L-Y |

*Underlined residues in cognate epitope are considered critical for recognition based on amino acid scanning. AP2S to KDM71 were identified using ScanProsite and CRA_b to SCCPC were identified using NCBI Protein Blast.*

**Supplemental Figures:**


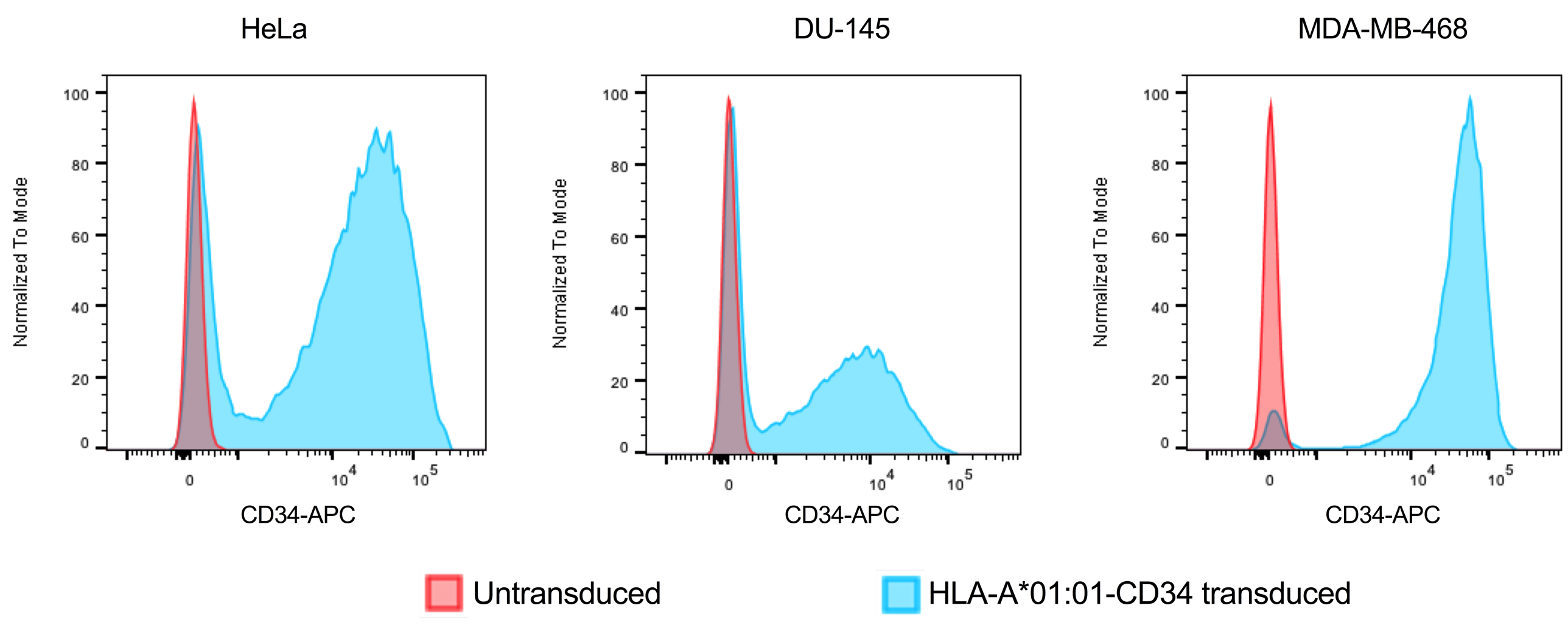


**Figure S1: Determination of HLA-A*01:01 expression in transduced cell lines by flow cytometry.**  Cell lines were transduced with a retroviral vector encoding HLA-A*01:01 and CD34. The cells were then bead-enriched for CD34 and transduction efficiency was assessed. Populations shown are gated on live, single, CD3+ cells.

**Figure S2: *CT83* expression levels differed in the cell lines used for in vivo experiments.**  (A) Sample images of the *CT83*+ cell lines 4156 and A375 by RNAScope are shown. Magnification is 20X. Nuclei were counterstained with DAPI (blue). (B) The frequency of *CT83*+ tumor cells was scored by an independent, blinded pathologist. N=10 for each cell line. (C) A375 tumors from mice that received 1×10^7^ UT-Ts and 1×10^7^ KK-LC-1 TCR-Ts were excised at day 9 and 56 post-treatment, respectively. cDNA from these tumors tumor cells was generated and tested for *CT83* expression by qRT-PCR. The error bars represent SD of two technical replicates. This experiment was performed once.


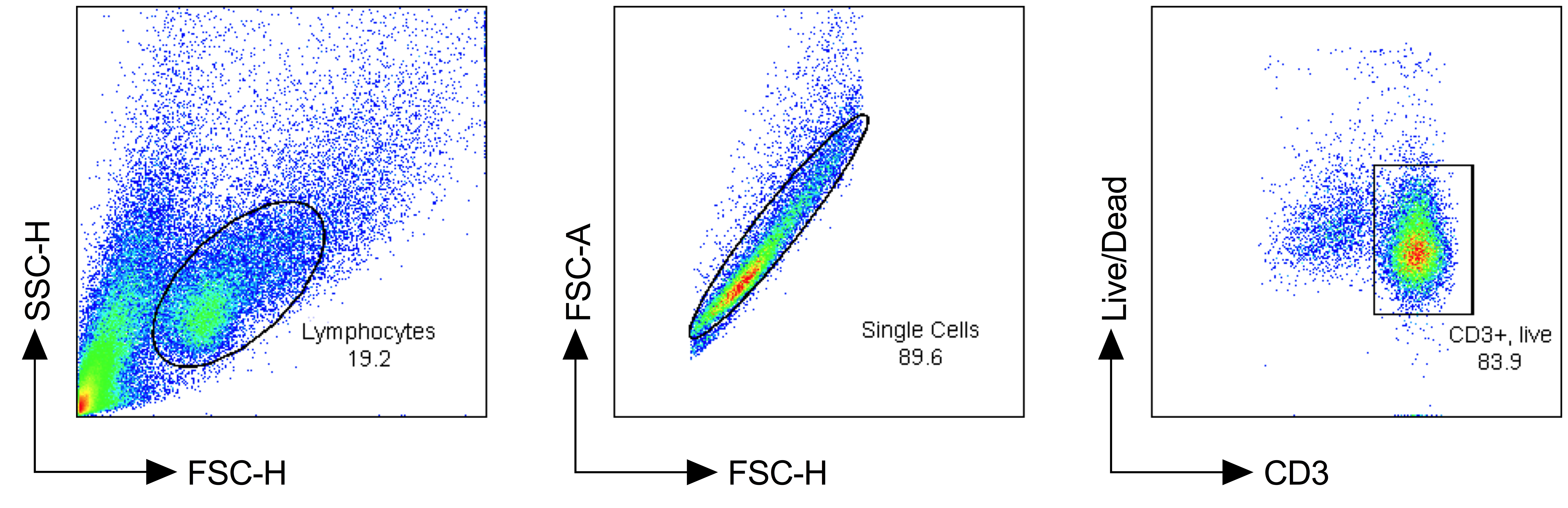


**Figure S3: Sample gating strategy for flow cytometry.**  For all flow cytometric analyses, before examining the population of interest, cells were first gated on lymphocytes using forward scatter (FSC) and side scatter (SSC). Next, doublets were excluded using FSC-height (H) and FSC-area (A). Finally, CD3+, live cells were isolated using propidium iodide (Sigma Aldrich).
